# Development of Head Space Sorptive Extraction Method for the Determination of Volatile Compounds in Beer and Comparison with Stir Bar Sorptive Extraction

**DOI:** 10.3390/foods9030255

**Published:** 2020-02-27

**Authors:** José E. Ruvalcaba, Enrique Durán-Guerrero, Carmelo G. Barroso, Remedios Castro

**Affiliations:** 1 Facultad de Ciencias Químicas, Universidad Autónoma de San Luis Potosí, Av. Dr. Manuel Nava #6, Zona Universitaria, 78210 San Luis Potosí, Mexico; joseleazar77@gmail.com; 2 Analytical Chemistry Department, Faculty of Sciences-IVAGRO, University of Cadiz, Agrifood Campus of International Excellence (ceiA3), Polígono Río San Pedro, s/n, Puerto Real, 11510 Cádiz, Spain; carmelo.garcia@uca.es (C.G.B.); remedios.castro@uca.es (R.C.)

**Keywords:** headspace sorptive extraction, beer, volatile compounds, stir bar sorptive extraction

## Abstract

A headspace sorptive extraction method coupled with gas chromatography–mass spectrometry (HSSE–GC–MS) was developed for the determination of 37 volatile compounds in beer. After optimization of the extraction conditions, the best conditions for the analysis were stirring at 1000 rpm for 180 min, using an 8-mL sample with 25% NaCl. The analytical method provided excellent linearity values (*R^2^* > 0.99) for the calibration of all the compounds studied, with the detection and quantification limits obtained being low enough for the determination of the compounds in the beers studied. When studying the repeatability of the method, it proved to be quite accurate, since RSD% values lower than 20% were obtained for all the compounds. On the other hand, the recovery study was successfully concluded, resulting in acceptable values for most of the compounds (80–120%). The optimised method was successfully applied to real beer samples of different types (ale, lager, stout and wheat). Finally, an analytical comparison of the optimised HSSE method, with a previously developed and validated stir bar sorptive extraction (SBSE) method was performed, obtaining similar concentration values by both methods for most compounds.

## 1. Introduction

Beer is one of the oldest and most widely consumed alcoholic beverages in the world, and the third most popular drink after water and tea [1]. Beer is usually made from malted barley, but other grains such as wheat, corn, or rice can also be used. During the brewing process, the fermentation of sugars from starch produces ethanol and carbon dioxide [2]. Most modern beers are brewed in the presence of hops, which add bitterness and aroma to the finished beer, as well as acting as a natural preservative and stabilising agent. Other possible flavourings that can be used in addition to or instead of hops are gruit (herbal mixture), herbs, or fruits.

The final aroma and taste of a particular beer is the result of hundreds of aromatically active compounds, which are produced during the course of the brewing process. However, the vast majority of the compounds are produced during the fermentation phase and are mainly metabolic intermediates or yeast by-products. Higher alcohols, esters and vicinal diketones, which determine the final quality of each beer, are some of the key compounds produced by the yeast [3]. While higher alcohols and esters are positive compounds that produce a pleasant aroma, vicinal diketones are often considered off-flavours. In addition, yeast metabolism generates other types of compounds, such as organic acids, sulphur compounds and aldehydes [3]. 

The aroma of the different beers is one of the most important aspects related to the final quality of the product. In addition, it is important to keep the off-flavours within certain limits in order to achieve a pleasant final aroma and thus obtain good consumer acceptance. It is, therefore, important to develop analytical methodologies that are sensitive, accurate, rapid and uncomplicated, and that are also capable of quantifying the volatile compounds responsible for each beer specific aroma. In the past decades, the development of automated and miniaturised sample preparation methods, which reduce or eliminate solvent consumption, has been a dominant trend in analytical chemistry. 

Stir bar sorptive extraction (SBSE) is a sample preparation technique developed by Baltussen et al. [4] and is a solvent-less enrichment technique by means of sorptive extraction. In 2000, one year after SBSE was developed, sorptive extraction was applied to headspace by Tienpont et al. [5] and Bicchi et al. [6] under the name of headspace sorptive extraction (HSSE). Both techniques are based on the sorption of the analytes into a thick layer of polydimethylsiloxane (PDMS) that covers a magnetic stirring bar with a glass cover. The analytes are extracted either by introducing the PDMS stirring bar directly into the liquid sample (SBSE) or by placing it in the headspace of the sample for a period of time (HSSE). The efficiency of the extraction process depends on the polarity of the analytes and is more efficient with compounds of medium–low polarity because of PDMS’s low polar character. However, due to the large amount of PDMS used for the stirring bars, both techniques present much higher recoveries than other similar ones (such as solid-phase micro extraction, SPME) for compounds with a low polarity [7].

In recent years, different applications of SBSE coupled with GC–MS for the aroma characterization of beers have appeared in the literature [8,9,10,11,12,13], but nevertheless, HSSE has been significantly less employed for this purpose. However, this latter technique has been successfully used for the determination of off-flavours in aged beers [14] and for the study of volatile compounds derived from hops, with similar results to those obtained when using SBSE [15]. Although immersion techniques are generally more sensitive, headspace extraction has the advantage that it reduces the risk of contamination and increases the lifetime of the stirring bar as well as being more representative of the aroma perceived by consumers [16].

The objective of this research is to develop a new solventless methodology for the analysis of volatile compounds in beers, employing HSSE. This is the first time that the extraction conditions for HSSE are optimised for the analysis of an extensive number of volatile compounds responsible for the aroma of beer and belonging to different chemical families. The optimised method was analytically validated and successfully applied to different types of beer (ale, lager, stout and wheat). A comparison with the SBSE method previously developed by this research group [13] has also been conducted, and it has been demonstrated that the results obtained by the two analysis techniques are generally comparable. So, with this new HSSE methodology, it is possible to characterise the aromatic profile of beers in a reliable way, employing a minimal amount of sample and decreasing the degradation of stirring bars. 

## 2. Materials and Methods 

### 2.1. Chemical and Reagents

A total of 37 volatile compounds from different chemical families were studied and are presented in Table 1 along with their retention times, chemical family and quantifying ions.

4-Methyl-2-pentanol (retention time: 21.9 min) and 2-octanol (retention time: 30.7 min) were used as internal standards and each volatile compound studied was referred to as one of the two standards (Table 1).

All the standards used in the study presented purity levels above 99% and were acquired from Sigma-Aldrich (St. Louis, MO, USA).

### 2.2. Stardards Preparation

Standard solutions of all the studied volatile compounds were prepared at a concentration of about 100 mg/L in a synthetic beer matrix (5% ethanol–water mixture). For calibration purposes, six concentration levels were prepared using the standard solutions in duplicate, in the range of 0.04 to 2000 µg/L, which allowed to determine the volatile compounds found in the real samples. For the recovery study, increasing concentrations in duplicate (20, 40, 100 and 200 µg/L) of the standards were added to a lager beer sample.

The internal standard solutions were prepared in synthetic beer matrices at a concentration of 2300 mg/L in the case of 4-methyl-2-pentanol and 104 mg/L in the case of 2-octanol. All the solutions were stored at 4 °C until their use.

### 2.3. Beer Samples

For the experimental design, the repeatability study and the recovery study, lager beer was used. Then, ten beers of different styles (4 lagers, 2 ales, 2 stouts and 2 wheats) were analysed according to the optimised HSSE method. All the beers used in the study were purchased from local markets and kept refrigerated at 4 °C until analysis. All the analyses were carried out in duplicate.

### 2.4. Head Space Sorptive Extraction

Once the method had been optimised, 8 mL of beer was placed in a 20-mL vial designed for headspace analysis, supplied by Gerstel (Mülheim an der Ruhr, Germany). Then, 25% salt (*w/v*), a small stirring bar and 16 µL of each internal standard were added. The extraction bar, commercially known as Twister^®^ (Gerstel), was placed in an adapted holder with a small opening in its bottom to keep it in the upper part of the vial. Then the vial was sealed by encapsulating it with an aluminium cap and a PTFE/silicone septum. The twisters used were made of 10 mm long and 0.5 mm thick PDMS. The vial was placed on an agitator plate at 1000 rpm for 180 min. When the extraction process was completed, the vial was opened and the twister was washed with distilled water for 20 seconds and then dried using paper and placed on a glass liner for its subsequent chromatographic analysis. No conditioning or cleaning of the twisters were performed after each analysis. 

### 2.5. Instrumentation

The sampling system consisted of a thermal desorption unit (TDS-2) equipped with multipurpose sampler (MPS) and a programmed temperature vaporization (PTV) cooled injector system (CIS-4) by Gerstel. The thermal desorption unit was operated in splitless mode. The desorption temperature was set up to climb from 40 to 300 °C at 60 °C/min and 10 minutes holding time, with a helium flow of 75 mL/min. The desorbed analytes were then cryofocused in the CIS using liquid nitrogen at −140 °C. The CIS was set up to climb from −140 to 300 °C at a 10 °C/s rate before GC–MS analysis. 

For the GC–MS analysis of the samples, a 7890 gas chromatography system coupled with a 5975C inert mass spectrometry detector (Agilent Technologies, Palo Alto, CA, USA) was employed. The capillary column was a DB-Wax (J&W Scientific, Folsom, CA, USA), 60 m × 250 μm × 0.25 μm. Helium as a carrier gas was maintained at 1 mL/min flow. The GC oven was started at 35 °C, held at that temperature for 10 min and then ramped up to 100 °C at a 5 °C/min rate, then the temperature was increased to 210 °C at a 3 °C/min rate and finally held at that temperature for 40 minutes. The compounds were identified by comparing the mass spectra obtained with those in Wiley 7N (Wiley Registry of Mass Spectral Data, 7th Edition, 2000, John Wiley & Sons, NJ, Hoboken, USA) and by comparing their retention times and mass spectra with commercial standards.

### 2.6. Comparative Study against Stir Bar Sorptive Extraction

For the comparative study against SBSE, four additional beers (one from each type) were analysed using HSSE and SBSE in duplicate. The testing conditions for SBSE were those developed in an earlier study by our research team [13]. A comparison was made between the concentration values obtained by HSSE and SBSE of all the compounds studied.

### 2.7. Statistic Tools

An experimental design was conducted to optimise the extraction conditions. The total chromatographic area obtained and the number of peaks were considered as the experimental response. A full factorial design 3^2^ was used to determine which factors had a significant effect on the response. The statistics program used to carry out this study was Statgraphic Centurion XVII (Statpoint Technologies, Inc., Warrenton, VA, USA).

The concentration data of the volatile compounds studied were subjected to analysis of variance (ANOVA) and a subsequent post-hoc analysis of means comparison (Tukey’s test) at a 5% significance level. The statistic application StatSoft GmbH, Hamburg, Germany was used to perform this statistical study.

## 3. Results and Discussion

### 3.1. Optimisation of the Extraction Conditions

Potential factors that may affect HSSE are sample amount, extraction time, agitation speed, salt addition, among others [16]. Although the extraction temperature is a parameter that may affect the process, it was not taken into account in this study. Other authors have indicated that, although an increase in temperature shortens the time required to reach equilibrium, it also increases the solubility of the analytes in water, which means that the amount extracted by the stirring bar may decrease [17]. As our study seeks to obtain maximum sensitivity rather than processing speed, the analyses were performed at room temperature.

Even though the sample volume normally has a positive effect on the extraction of volatile compounds in food samples by PDMS, i.e., the larger the sample volume, the more efficient the extraction [18,19,20,21], in the specific case of HSSE, other researchers have concluded that sample volume is not significant with respect to extraction [16,22]. In our case, the maximum volume allowed by the experimental device was taken, namely, 8 mL. In any case, the sample volume used for this technique is considerably smaller than that used for SBSE, where the optimised volume was 50 mL [13]. 

Adding salt has been proven to be an important variable to improve the extraction of volatile compounds from beer by SBSE [13] and from other water matrices such as vinegar [18] or orange juice [23]. The addition of salt to the medium changes the ionic strength of the medium, which in turn affects the relative polarity of the compounds and thus the extraction of the compounds by the (low-polar) PDMS polymer. Consequently, a value of 25% was set for our study. Other authors employed similar values of salt for the extraction of volatile compounds in beers by SPME [24].

**Figure 1:**
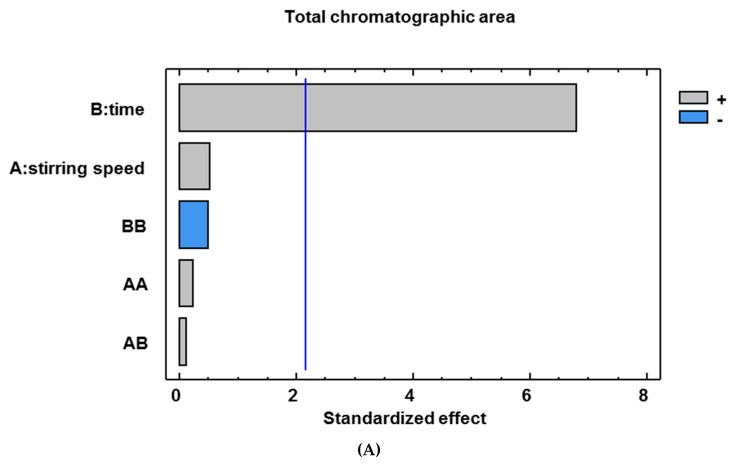

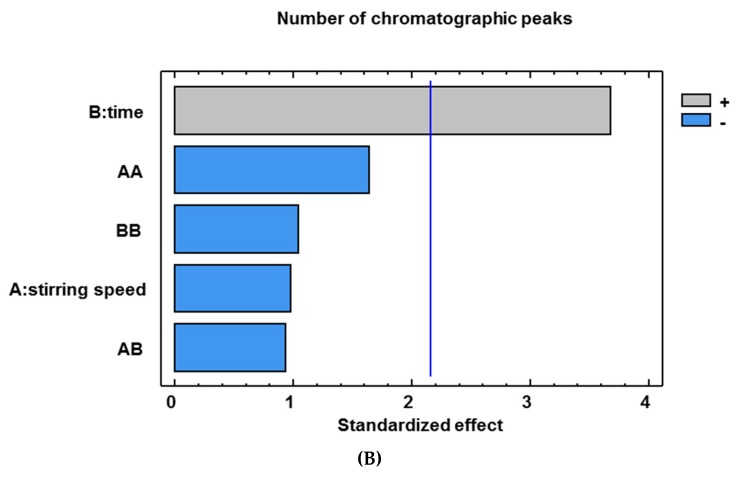
Pareto charts for total chromatographic area (A) and number of chromatographic peaks (B) as experimental responses.

For the study of both variables “extraction time” and “stirring speed” a factorial 3^2^ design was carried out. Ten experiments were performed (in duplicate) by modifying their extraction time (30–180 min) and stirring speed (500–2000 rpm) in order to observe their influence on the experimental responses “total chromatographic area” and “number of chromatographic peaks”. Both experimental responses are related to the total amount of volatile compounds extracted. The data were evaluated by ANOVA at a 5% significance level. The main effects and interactions observed can be seen in the Pareto charts in Figure 1.

Regarding both the number of chromatographic peaks as well as the total area, it can be observed that extraction time is the only factor with a significant positive effect (*p* < 0.05; Figure 1). Therefore, it can be deduced from this fact that longer extractions result in the extraction of a greater number of compounds and a greater amount of the same. The optimum value proposed by our design was over 200 minutes, but for operational reasons, it was set at 180 minutes.

Stirring speed did not reveal any significant influence and was therefore set at the intermediate value of 1000 rpm.

### 3.2. Analytical Validation of the Optimised Method

Thirty-seven volatile compounds were studied for calibration, employing the optimal conditions that had been obtained after the optimisation process. Table 2 shows the outcome of the calibration for the compounds studied. As can be seen, virtually all the determination coefficients obtained (R^2^) were higher than 0.99. 

The limits of detection (LOD) and the limits of quantification (LOQ) were calculated based on the calibration curves according to the following formulae:

𝐿𝑂𝐷 = 3𝑆𝐼/𝑏
𝐿𝑂𝑄 = 10𝑆𝐼/𝑏
SI = standard deviation of the intercept of the regression line
b = slope from the regression line

Most of the compounds studied presented acceptable LOD and LOQ values and were low enough to be quantified in beer samples. However, some of the results obtained in this case were slightly higher than those obtained using the SBSE method [13]. In addition, a total of 52 compounds had been studied using this method, compared to the 37 compounds studied in this particular case. This result should be considered logical since, when a headspace method is used, sensitivity decreases compared to that of submerged extractions, which means that the number of compounds detected in the real samples may be lower. However, the results obtained in headspace are typically more representative of what the consumer perceives by smelling the beer. In addition, HSSE is usually more sensitive than other headspace methodologies such as HS-SPME because of the higher amount of polymer available, but extractions are usually longer. Other authors quantified 19 volatile compounds in beers employing HS-SPME, with 30 minutes of extraction [25]. Giannetti et al. [26] performed SPME extractions of 10 minutes, but no quantification was carried out.

The accuracy of our method was evaluated through inter-twister precision and inter-day precision. Inter-twister precision was evaluated by extracting five replicates from a single lager sample on the same day, using different twisters. The repeatability of the method on different days (inter-day precision) was evaluated by extracting five beer samples on five consecutive days, using the same twister for the extractions. The coefficients of variation of the detected compounds were calculated (Table 2). The results obtained were lower than 20%, which are generally accepted values for this type of technique and thus corroborate the high precision level of the methodology employed. Similar values were obtained for the determination of volatile compounds in beers when SBSE was the technique used [13]. Other methodologies such as SPME also provide similar values of precision [27].

In addition, the recovery values of the compounds studied in a larger sample were calculated after different concentration values were added (Table 2). Of the 37 compounds studied, only seven were outside the generally accepted values, with recovery values outside the range 80–120%, even though some of them presented values close to this range (linalool: 77.16%; β-damascenone: 77.96%; octanoic acid: 130.67%). On the other hand, the recovery values for three other compounds could not be calculated (Table 2) since they did not show a linear correlation between the added concentrations and their experimental responses. Other authors [24] obtained better values of recovery for these compounds employing SPME, but with polyacrylate (PA) as extracting polymer and they found better responses when they compared PA to PDMS fibres. The almost single current use of PDMS for HSSE could be a limitation due to the low polarity of this polymer.

Finally, in order to demonstrate the applicability of the optimised method, different real samples of different types of beer (4 lager, 2 ale, 2 stout and 2 wheat) were analysed in duplicate. The results obtained are shown in Table 3. This table also presents the significant differences between the compounds’ concentrations following the application of the Tukey test (α = 0.05). As can be seen, the compound with the highest concentration in all the types of beer was 3-methyl-1-butanol, followed by ethyl octanoate, ethyl hexanoate, isopentyl acetate, nerolidol, phenylethyl acetate and octanoic acid. Most of these compounds have demonstrated to be odour-active compounds for the aroma of beers [28]. Similar results had been found in previous studies where different extraction techniques had been used [8,13,25,27,29]. However, other compounds such as benzaldehyde, guaiacol, linalool, hexanoic acid 2-phenylethyl ester or heptanol were only identified in some of the beer types. Also, volatile compounds such as hexanal or nonanal, normally considered as off-flavours [14], were not detected in any of the samples studied. Other authors [14] also found no detectable concentrations of hexanal in the samples of beers that had been treated with light and heat. As can be seen, our method has proven to be suitable for the analysis of volatile compounds in beers.

### 3.3. Comparative Study against SBSE

To thoroughly test whether the optimised HSSE methodology was providing similar results to the SBSE methodology, a duplicate analysis of four beers (one of each type studied) was carried out using both methodologies. The concentrations obtained by both methodologies were plotted against each other and a linear regression was performed so as to obtain a line equation ([SBSE] = a [HSSE] + b) for each of the compounds studied. The slope of the calculated lines indicates the level of similarity of the data obtained, where a slope value equal to 1 corresponds to a perfect similarity between both methodologies. The data obtained from the linear regression are shown in Table 4. In some cases, the analysis was not feasible since some compounds were not detected by both techniques when applied to the same beer samples. It can be seen that most of the compounds that were determined presented similar concentration values by both HSSE and SBSE. Therefore, it has been demonstrated that the HSSE methodology developed in this study provides similar results to those obtained by the SBSE methodology previously developed by this research group [13]. 

## 4. Conclusions

It is clear that HSSE is an appropriate method to determine the different types of volatile compounds in beers. The results that have been obtained are similar to those achieved by other more widely accepted techniques, such as SBSE. However, HSSE presents some advantages, such as a significantly lower degradation of the stirring bars when compared to the submersion method used in SBSE. Furthermore, the volume of the samples is considerably reduced and also the aroma detected by HSSE is considered as more representative of what consumers perceive when they smell a particular beer. Even though it is true that sensitivity levels are slightly lower, the detection and quantification limits that have been obtained allow us to determine a significant number of the volatile compounds that are present in beer samples from different types. 

## Figures and Tables

**Table 1:** Volatile compounds studied, retention times, internal standard employed, chemical family and MS fragment employed for the quantification.

Volatile Compound	Retention Time (min)	Internal Standard *	Chemical Family	SIM
isobutyl acetate	15.16	A	branched alkyl ester	43
ethyl butyrate	16.33	A	ethyl ester (linear chain)	43
ethyl isovalerate	17.70	A	branched alkyl ester	88
hexanal	18.20	A	aldehyde (linear chain)	44
ethyl pentanoate	20.11	B	ethyl ester (linear chain)	88
isopentyl acetate	20.11	B	branched alkyl ester	43
3-methyl-1-butanol	23.38	A	alcohol (branched chain)	55
ethyl hexanoate	24.35	B	ethyl ester (linear chain)	88
hexyl acetate	25.65	A	linear alkyl acetate ester	43
octanal	26.89	A	aldehyde (linear chain)	41
6-methyl-5-hepten-2-one	27.85	A	ketone (linear)	43
ethyl heptanoate	27.89	B	ethyl ester (linear chain)	88
1-hexanol	28.25	A	alcohol (linear chain)	56
heptyl acetate	29.28	B	linear alkyl acetate ester	43
nonanal	30.07	B	aldehyde (linear chain)	57
ethyl octanoate	31.56	A	ethyl ester (linear chain)	88
heptanol	31.94	A	alcohol (linear chain)	70
isopentyl hexanoate	32.96	A	branched alkyl ester	70
octyl acetate	32.99	B	linear alkyl acetate ester	43
benzaldehyde	34.49	A	aldehyde (aromatic)	77
linalool	35.22	B	alcohol (alkene chain)	41
isobutyric acid	35.69	B	carboxylic acid (branched chain)	41
octanol	35.69	B	alcohol (linear chain)	41
2,3-dihydrobenzofurane	37.42	A	cyclic ether	91
ethyl decanoate	39.02	B	ethyl ester (linear chain)	88
benzenoic acid ethyl ester	39.83	A	aromatic alkyl ester	105
1-decanol	43.16	B	alcohol (linear chain)	41
phenylethyl acetate	45.14	B	aromatic alkyl ester	104
β-damascenone	45.97	A	ketone (cyclic)	69
guaiacol	46.24	B	cyclic ether	81
ethyl dodecanoate	46.35	A	ethyl ester (linear chain)	88
benzopropanoic acid ethyl ester	47.46	B	aromatic alkyl ester	104
hexanoic acid 2-phenylethyl ester	47.46	B	aromatic alkyl ester	104
isobutyric acid phenethyl ester	48.21	A	aromatic alkyl ester	104
nerolidol	52.94	B	alcohol (alkene chain)	41
octanoic acid	52.94	A	carboxylic acid (linear chain)	60
β-phenylethyl-2-methylbutyrate	56.78	A	aromatic alkyl ester	104

* A: 4-methyl-2-pentanol; B: 2-octanol. SIM: selected ion monitoring.

**Table 2:** Analytical features of the headspace sorptive extraction (HSSE) method for the studied compounds.

Volatile Compound	Slope	Intercept	*R* ^2^	LOD (µg/L)	LOQ (µg/L)	Recovery (%)	Inter-tw Precision * (%)	Inter-Day Precision ** (%)
isobutyl acetate	0.0017	0.0728	0.9998	25.48	84.95	96.22	15.53	9.12
ethyl butyrate	0.0006	0.0150	0.9980	2.77	9.26	110.44	13.70	8.19
ethyl isovalerate	0.0010	0.0015	0.9999	0.30	1.02	101.89	4.65	7.45
hexanal	0.00008	0.0066	0.9902	12.76	42.54	100.39	10.52	19.43
ethyl pentanoate	0.0030	0.0077	0.9998	0.85	2.84	104.02	4.86	13.81
isopentyl acetate	0.0096	0.3258	0.9970	19.63	65.45	-	9.98	5.91
3-methyl-1-butanol	0.00003	0.0053	0.9961	68.16	227.20	-	16.47	9.51
ethyl hexanoate	0.0065	0.2460	0.9979	10.80	36.01	103.18	9.85	5.32
hexyl acetate	0.0071	−0.0148	0.9990	0.96	3.21	105.25	5.71	6.03
octanal	0.0016	−0.0005	0.9991	1.38	4.60	103.00	17.35	17.54
6-methyl-5-hepten-2-one	0.0014	0.0047	0.9993	1.85	6.16	132.87	12.66	8.11
ethyl heptanoate	0.0115	−0.0018	0.9980	1.10	3.66	94.84	9.61	2.67
1-hexanol	0.0001	0.0010	0.9997	0.93	3.12	90.88	13.05	11.30
heptyl acetate	0.0231	0.0055	0.9994	0.25	0.85	110.07	6.14	17.38
nonanal	0.0038	0.0438	0.99905	7.68	25.62	113.91	4.73	16.39
ethyl octanoate	0.0052	0.0222	0.9990	4.18	13.94	86.06	12.30	5.67
heptanol	0.0002	0.0016	0.9978	10.80	36.01	81.26	-	-
isopentyl hexanoate	0.0050	0.0002	0.9992	0.01	0.05	93.60	12.73	12.24
octyl acetate	0.0286	−0.1174	0.9989	0.95	3.16	88.38	14.42	10.14
benzaldehyde	0.0001	0.0050	0.9962	0.35	1.16	104.75	6.78	3.16
linalool	0.0021	0.0220	0.9990	2.64	8.80	77.16	11.73	15.30
isobutyric acid	0.0015	0.0157	0.9997	1.59	5.30	87.10	13.78	4.33
octanol	0.0015	0.0116	0.9996	0.72	2.41	86.57	6.43	16.03
2,3-dihydrobenzofurane	0.0013	−0.0031	0.9708	0.16	0.54	90.13	-	-
ethyl decanoate	0.0060	0.0283	0.9994	0.31	1.04	98.03	8.99	9.69
benzenoic acid ethyl ester	0.0026	−0.0137	0.9989	0.24	0.82	81.76	11.98	10.26
1-decanol	0.0019	0.0283	0.9994	6.42	21.41	<60	7.21	10.65
phenylethyl acetate	0.0020	0.1798	0.9914	5.95	19.85	111.73	9.30	6.23
β-damascenone	0.0020	−0.0091	0.9982	1.09	3.63	77.96	11.52	7.64
guaiacol	0.00004	0.0029	0.9907	1.69	5.65	-	-	-
ethyl dodecanoate	0.0003	0.0018	0.9987	0.62	2.07	82.29	17.59	19.34
benzopropanoic acid ethyl ester	0.0016	0.0030	0.9997	1.23	4.11	96.74	16.90	18.59
hexanoic acid 2-phenylethyl ester	0.0018	0.0001	0.9995	0.78	2.60	88.61	-	-
isobutyric acid phenethyl ester	0.0007	−0.0043	0.9953	2.56	8.53	<60	14.38	10.18
nerolidol	0.0003	0.0214	0.9938	15.31	51.03	96.28	14.49	17.07
octanoic acid	0.0003	0.0134	0.9997	11.44	38.14	130.67	13.26	15.33
β-phenylethyl-2-methylbutyrate	0.0009	0.00003	0.9993	0.36	1.21	<60	11.78	17.15

* Coefficient of variation (%) calculated for 5 replicates using 5 different twisters the same day; ** coefficient of variation (%) calculated for 5 replicates in 5 different days using the same twister; LOD: limit of detection; LOQ: limit of quantification.

**Table 3:** Concentrations (µg/L) determined of volatile compounds in beers by HSSE–GC–MS.

Beer Sample	Lager (*N* = 8)		Wheat (*N* = 4)		Stout (*N* = 4)		Ale (*N* = 4)	
Volatile Compound	Mean	SD	Mean	SD	Mean	SD	Mean	SD
isobutyl acetate	<LOQ a	-	128.05 b	67.07	<LOQ a	-	<LOQ a	-
ethyl butyrate	107.85 a	25.26	88.81 a	8.32	103.94 a	29.18	145.21 a	44.62
ethyl isovalerate	4.08 a	4.59	<LOQ	-	4.01 a	2.46	2.04 a	0.96
hexanal	ND	-	ND	-	ND	-	ND	-
ethyl pentanoate	13.24 a	7.66	40.78 b	18.46	13.74 a	1.99	6.33 a	4.96
isopentyl acetate*	2.61 b	0.45	4.31 c	0.87	0.75 a	0.26	1.47 a	0.78
3-methyl-1-butanol*	51.32 a	12.37	39.82 a	17.15	38.94 a	8.18	64.89 a	24.07
ethyl hexanoate	307.14 a	259.04	64.90 a	8.01	284.65 a	247.13	361.33 a	97.23
hexyl acetate	7.33 b	3.07	6.86 ab	1.46	3.43 a	0.75	3.74 ab	0.87
octanal	5.54 a	3.02	<LOQ	-	8.39 a	6.14	5.02 a	4.07
6-methyl-5-hepten-2-one	6.36 a	5.12	<LOQ	-	26.22 a	27.64	12.72 a	12.32
ethyl heptanoate	<LOQ	-	<LOQ	-	12.25 a	12.74	6.02 a	4.80
1-hexanol	11.26 a	2.97	12.45 a	9.50	56.79 a	57.27	33.79 a	19.39
heptyl acetate	<LOQ	-	1.13 a	0.06	3.41 b	0.23	2.13 ab	1.79
nonanal	ND	-	ND	-	ND	-	ND	-
ethyl octanoate	139.08 a	52.82	103.08 a	20.90	529.03 a	514.51	762.58 a	655.03
heptanol*	ND	-	ND	-	1.25 a	1.09	4.12 b	0.71
isopentyl hexanoate	0.35 a	0.21	0.53 a	0.12	0.49 a	0.65	1.35 b	0.26
octyl acetate	5.49 b	0.62	5.25 b	0.21	4.16 a	0.04	4.37 a	0.21
benzaldehyde	7.87 a	3.12	ND	-	ND	-	5.80 a	2.32
linalool	<LOQ	-	ND	-	100.13 a	3.45	106.42 a	26.66
isobutyric acid	6.05 a	3.28	5.66 a	0.11	<LOQ	-	17.81 b	3.00
octanol	5.47 a	2.55	8.64 a	0.27	6.76 a	0.41	15.83 b	8.10
2,3-dihydrobenzofurane	3.48 a	1.66	3.23 a	0.98	3.01 a	0.62	ND	-
ethyl decanoate	50.07 a	47.31	57.11 ab	5.72	66.44 ab	44.73	154.61 b	87.21
benzenoic acid ethyl ester	5.87 a	0.49	5.95 a	0.25	6.43 ab	0.53	7.17 b	0.46
1-decanol	47.28 a	23.98	<LOQ	-	64.99 a	46.59	<LOQ	-
phenylethyl acetate	360.93 b	98.68	378.90 b	26.03	31.31 a	20.49	141.57 a	76.76
β-damascenone	4.63 a	0.10	4.84 a	0.12	7.37 b	0.11	6.99 b	1.91
guaiacol	22.92 a	22.80	ND	-	69.46 b	5.14	ND	-
ethyl dodecanoate	17.38 a	19.98	2.36 a	0.51	19.13 a	11.48	11.99 a	11.93
benzopropanoic acid ethyl ester	8.51 a	3.12	4.30 a	2.40	5.77 a	3.39	15.17 b	4.94
hexanoic acid 2-phenylethyl ester	<LOQ	-	ND	-	4.87 a	5.03	3.63 a	2.13
isobutyric acid phenethyl ester	12.20 ab	2.73	9.09 a	0.79	10.26 a	2.47	15.58 b	3.57
nerolidol	380.29 ab	179.77	172.73 a	54.20	192.11 a	119.94	621.57 b	174.28
octanoic acid	199.88 ab	118.62	88.90 a	10.53	95.84 a	68.18	288.87 b	69.42
β-phenylethyl-2-methylbutyrate	3.83 a	4.76	2.69 a	1.20	<LOQ	-	<LOQ	-

* mg/L; SD: standard deviation; ND: not detected; <LOQ: below limit of quantitation; For each compound, different letters indicate significant differences according to Tukey’s test (α = 0.05).

**Table 4:** Parameters of the linear regression obtained by representing concentration values determined with HSSE against concentration values determined with SBSE: [SBSE] = slope [HSSE] + intercept.

Volatile Compound	Slope	Intercept	*R* ^2^
isobutyl acetate	0.9069	6.2751	0.9989
ethyl butyrate	1.2508	−18.6980	0.9473
ethyl isovalerate	1.0774	-0.1628	0.9911
hexanal	-	-	-
ethyl pentanoate	1.1131	−0.8687	0.9964
isopentyl acetate	1.0094	−37.4939	0.9998
3-methyl-1-butanol	0.9250	7651.0458	0.8338
ethyl hexanoate	0.9942	0.3938	0.9954
hexyl acetate	1.9794	11.1685	0.9593
octanal	0.9057	0.7722	0.7429
6-methyl-5-hepten-2-one	1.0106	0.0371	0.9999
ethyl heptanoate	0.9804	0.0140	0.9994
1-hexanol	1.0336	−0.3775	0.9795
heptyl acetate	0.9967	0.0030	0.9999
nonanal	-	-	-
ethyl octanoate	0.9909	−0.5271	0.9864
heptanol	-	-	-
isopentyl hexanoate	1.6954	21.1004	0.2844
octyl acetate	0.9503	0.1023	0.9985
benzaldehyde	0.8895	0.6306	0.9999
linalool	1.1181	1.1259	0.9746
isobutyric acid	-	-	-
octanol	0.9963	0.4574	0.9221
2,3-dihydrobenzofurane	-	-	-
ethyl decanoate	1.1743	−8.3197	0.9636
benzenoic acid ethyl ester	1.0740	−1.6923	0.5956
1-decanol	0.9603	0.7992	0.9963
phenylethyl acetate	1.0524	39.9797	0.8937
β-damascenone	-	-	-
guaiacol	1.0057	0.3731	0.9999
ethyl dodecanoate	1.0780	−1.1354	0.9565
benzopropanoic acid ethyl ester	0.9646	0.0722	0.9950
hexanoic acid 2-phenylethyl ester	0.9977	0.0395	0.9976
isobutyric acid phenethyl ester	0.1649	−0.2036	0.5740
nerolidol	1.0683	175.1769	0.6893
octanoic acid	-	-	-
β-phenylethyl-2-methylbutyrate	1.0219	−0.1420	0.9898
